# Can Melatonin Improve the Osteopenia of Perimenopausal and Postmenopausal Women? A Meta-Analysis

**DOI:** 10.1155/2019/5151678

**Published:** 2019-04-07

**Authors:** Tingting Bao, Liuting Zeng, Kailin Yang, Yuehua Li, Fengying Ren, Yulong Zhang, Ziren Gao

**Affiliations:** ^1^Beijing University of Chinese Medicine, Beijing 100029, China; ^2^Xiyuan Hospital, China Academy of Chinese Medical Sciences, Beijing, China; ^3^Hunan University of Chinese Medicine, Changsha, Hunan, China

## Abstract

**Objective:**

To assess the effectiveness and safety of melatonin for perimenopausal and postmenopausal women with osteopenia.

**Methods:**

In this meta-analysis, data from randomized controlled trials were obtained to assess the effects of melatonin versus placebo or western medicine in perimenopausal and postmenopausal women with osteopenia. The study's registration number is CRD42018086238. The primary outcomes included bone mineral density (BMD) and T-score.

**Result:**

From 551 articles retrieved, three trials involving 121 patients were included. Due to the high-to-substantial heterogeneity (BMD: I^2^=96.9%, P=0.000; T-score: I^2^=74.9%, and P=0.019), the statistical analysis of BMD and T-score was abandoned. A systematic review was undergone for the two outcomes. Compared with the control group, melatonin may increase osteocalcin (WMD 4.97; 95% CI 3.14, 6.79; P < 0.00001).

**Conclusion:**

Based on current evidence, melatonin might be used as a safe nutritional supplement to improve bone density in perimenopausal and postmenopausal women, but its efficacy needs to be further affirmed.

## 1. Introduction

Osteopenia is a disorder of the skeletal system characterized by weakened bone resulting from the rate of bone resorption that surpasses the rate of bone formation [1]. It is defined as below normal bone density but above the bone density of osteoporosis, with a T-score -1 to -2.5 [1]. Osteopenia develops silently; and if not diagnosed and treated in time, it can result in osteoporosis and future fracture [1]. Similar to osteoporosis, osteopenia tends to occur in perimenopausal and postmenopausal women, mostly women over the age of 60 [1, 2]. It is manifested as deterioration of bone tissue and destruction of bone structure sometimes [3]. For such skeletal system, the mechanism of most treatment regimens (such as bisphosphonates, parathyroid hormone, raloxifene, calcitonin, and strontium ranelate) to date is to reduce bone resorption by inhibiting osteoclasts, thereby increasing bone density and improving bone bearing capacity [2, 4–6]. Although these are currently accepted treatment options, their adverse events affect their efficacy, such as the increased risk of the jaw necrosis and subtrochanteric or femoral shaft fractures [7–10], hot flashes, deep venous thrombosis [11], and osteosarcoma [12]. These adverse events may affect the compliance and lower the resorption, leading to the reduced drug efficacy [13]. These findings suggest the need to develop new alternatives to prevent or reverse bone loss.

Melatonin, as an endogenous factor secreted by the pineal [14], is considered as another alternative therapy for improving bone health [15, 16]. Melatonin can microregulate the bone formation and absorption through the dual action on osteoblasts and osteoclasts [16, 17]. In addition, the level of melatonin drops rapidly after menopause and is inversely related to age [18]. Hence, the occurrence of perimenopausal and postmenopausal osteoporosis may be related to the decrease of melatonin [19]. In a clinical study of perimenopausal women, the balance between bone‐resorbing osteoclasts and bone‐forming osteoblasts was restored by supplementation of melatonin (3 mg) for 6 months [15]. Animal and cell studies showed that melatonin can not only increase bone alkaline phosphatase levels, mineralization, and bone mass [20–22] but also promote new bone growth and osseointegration [23–25]. Thus, melatonin may be a new alternative for perimenopausal and perimenopausal women with osteopenia. Meanwhile, although several basic and clinical researches have shown the effect of melatonin on bone density, there is currently no meta-analysis to assess its efficacy on osteopenia. Therefore, the purpose of this meta-analysis was to evaluate the efficacy and safety of melatonin for perimenopausal and postmenopausal women with osteopenia.

## 2. Why It Is Important to Do the Review

Although melatonin has theoretical benefits for perimenopausal and postmenopausal women with osteopenia [16–25] and several clinical trials also evaluated the efficacy of melatonin by comparing it with placebo [15, 26–28], to our knowledge, there is no systematic review assessing the efficacy of melatonin on perimenopausal and postmenopausal women with osteopenia. Therefore, this meta-analysis summarizes available evidence from RCTs about the role of melatonin in perimenopausal and postmenopausal women with osteopenia.

## 3. Materials and Methods

### 3.1. Protocol

This meta-analysis was conducted based on a predefined protocol registered on PROSPERO (CRD42018086238) following the PRISMA-guidelines s (see Supplementary Materials) [29].

### 3.2. Search Strategy and Selection Criteria

The Chinese Science and Technology Periodical Database (VIP), Chinese Biomedical Database (CBM), PubMed, Web of Science, ClinicalTrials, Cochrane Library (until Issue 1, 2018), the China National Knowledge Infrastructure Databases (CNKI), EMBASE, Medline Complete, and Wan Fang Database were searched from their inception in January, 2018 [30].

Studies meeting the inclusion criteria were included in this review: (1) participants: women with perimenopausal or postmenopausal osteopenia; (2) intervention: melatonin with no limits on the type, dose, frequency, and so on; (3) comparisons: blank, placebo, and western medicine; (4) outcomes: primary outcomes: BMD, T-score; secondary outcomes: osteocalcin; (5) study type: randomized controlled trials (RCTs) without limits on the way to randomization generation, blinding, or publishing language [30].

### 3.3. Data Analysis

All studies were reviewed and selected independently by four reviewers (Tingting Bao, Liuting Zeng, Ziren Gao, and Yulong Zhang). The search strategy for PubMed was present in Table 1. The data were extracted independently by three reviewers (Tingting Bao, Liuting Zeng, Yuehua Li, and Fengying Ren) and any discrepancies among the reviewers were resolved by consensus among all six reviewers (Tingting Bao, Liuting Zeng, Yuehua Li, Ziren Gao, Yulong Zhang, and Fengying Ren).

The risk of bias was assessed using the risk of bias assessment tool by the Cochrane Handbook for Systematic Reviews of Interventions, version 5.1.0 [30, 31]. Three reviewers (Tingting Bao, Liuting Zeng, and Yuehua Li) independently performed this, and any discrepancies among the three reviewers were resolved by consensus among all six reviewers (Tingting Bao, Liuting Zeng, Yuehua Li, Ziren Gao, Yulong Zhang, and Fengying Ren). The figure and table of risk of bias were drawn by RevMan 5.3.

The statistical analyses were carried out using Stata SE version 15. The dichotomous variable measure was summarized by risk ratio (RR) with a 95% confidence interval (CI). The continuous outcomes underwent meta-analysis using mean differences (MD) and 95% CI. Heterogeneity among studies was assessed using Cochrane's Q and I^2^ statistic [30, 32]. When P>0.1,* I*2<50%, we used a fixed effect model; when P<0.1, I2>50%, we would explore the reasons for heterogeneity, perform the subgroup analysis, and use a random effect model.

## 4. Results

### 4.1. Results of the Search

**Figure 1:**
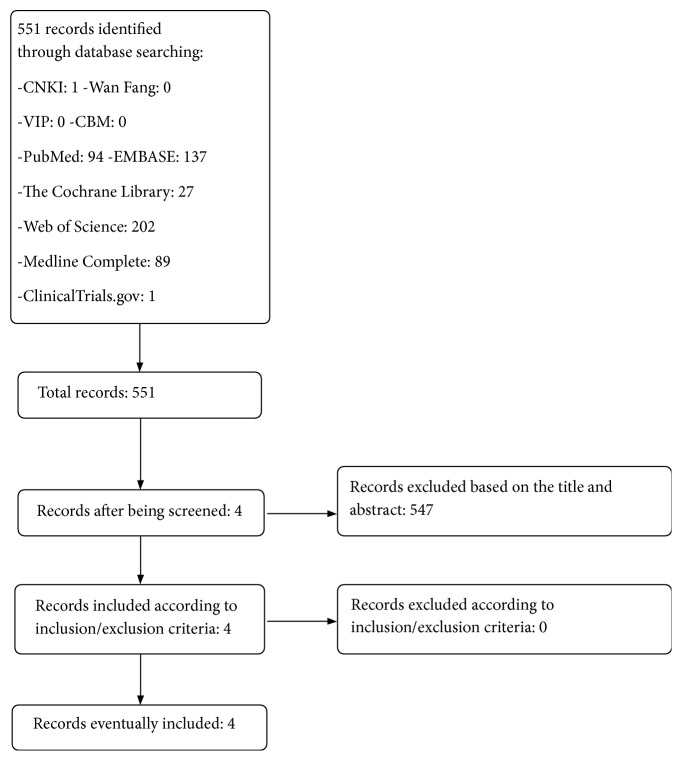
Flow diagram of searching and article selection.

Five hundred and fifty-one (551) articles were identified after initial search. Five hundred and forty-seven (547) articles were excluded based on the title and abstract. Finally, zero (0) records were excluded due to the exclusion criteria and 4 were included due to the inclusion criteria (Figure 1).

### 4.2. Description of Included Trials

Three RCTs (four records) with 121 participants met the inclusion criteria. There are two records data [26, 27] derived from the same clinical trial, so we counted them as one RCT (Amstrup 2015 [26, 27]). Study characteristics are presented in Table 2; the oral supplement composition is described in Table 3.

### 4.3. Risk of Bias of Included Studies

**Figure 2:**
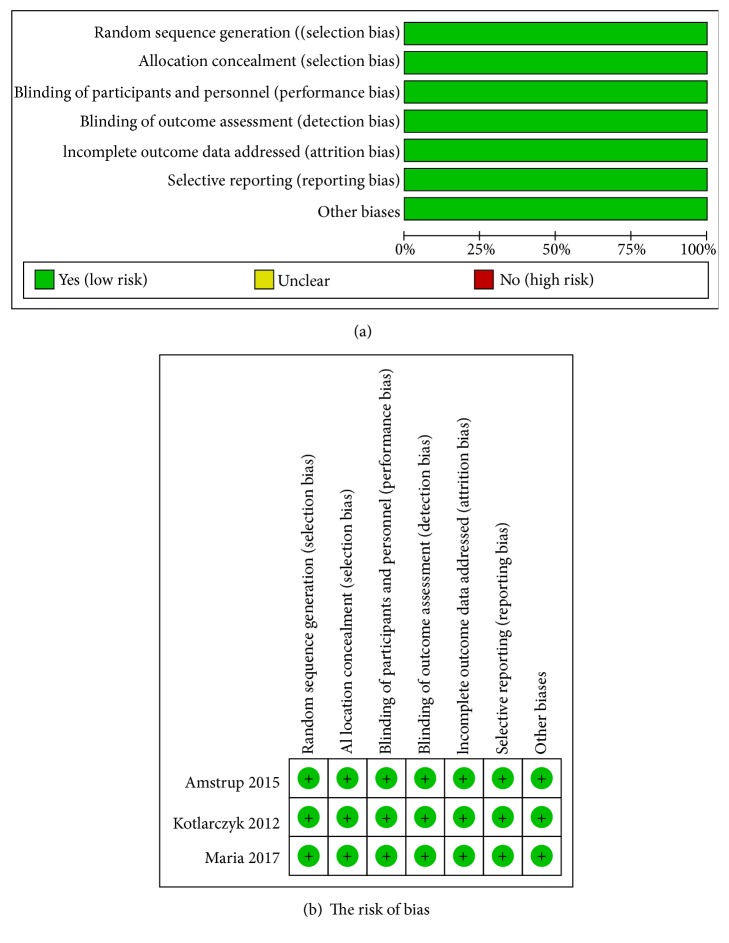


The summary and graph of risk of bias ware shown in Figure 2

#### 4.3.1. Sequence Generation

All RCTs described their randomization procedures: Amstrup 2015 [26, 27] utilized a restricted block randomization procedure, while Kotlarczyk 2012 [15] and Maria 2017 [28] used the computer-generated randomization scheme. Thus, all three RCTs were thought to have low risks of bias.

#### 4.3.2. Allocation Concealment

Amstrup 2015 [26, 27], Kotlarczyk 2012 [15], and Maria 2017 [28] used identical tablets or capsules, which were thought to be the acceptable methods of allocation concealment. Hence, these RCTs were rated as having low risks of bias.

#### 4.3.3. Blinding

For participant and outcome assessment blinding, all studies used blinding; thus, we gave a low risk of bias for all.

#### 4.3.4. Incomplete Outcome Data

The missing outcome data of Maria 2017 [28] balanced in numbers across intervention groups with similar reasons for missing data across groups. Amstrup 2015 [26, 27] and Kotlarczyk 2012 [15] used intention-to-treat analysis to analyze the missing data. Therefore, these RCTs were rated as having low risks of bias.

#### 4.3.5. Selective Reporting

All RCTs [15, 26–28] provided their protocols, and all of the study's prespecified outcomes that are of interest in the review had been reported in the prespecified way; their risks of bias were low.

#### 4.3.6. Other Potential Bias

Other sources of bias were not observed in 8 RCTs; therefore, the risks of other biases of the RCTs were low.

### 4.4. Primary Outcomes

#### 4.4.1. Bone Mineral Density

Two RCTs [26–28] reported the changes of BMD at the end of treatment. Maria 2017 [28] measured bone density by dual-energy X-ray absorptiometry (DXA), while Amstrup 2015 [26, 27] measured it by DXA and quantitative computed tomography (QCT). Meanwhile, Maria's findings are based on comparison of combination therapy with placebo. Due to the high heterogeneity (Chi^2^=64.03, Tau^2^=14.13, I^2^=96.9%, and P=0.000), the statistical analysis was abandoned according to the Cochrane Handbook for Systematic Reviews of Interventions [31]. Amstrup 2015 [26, 27] found that patients in the melatonin group had an increased aBMD (measured by DXA) in the femoral neck [1 mg melatonin group (P = 0.55); 3 mg melatonin group (P < 0.01)] and an increased vBMD (measured by QCT) in the lumbar spine [3 mg melatonin group (P = 0.04)]; in particular, the increase of aBMD in the femoral neck was dose dependent (P < 0.05). Maria 2017 [28] found that the difference between trial group and placebo group was statistically significant in the left femoral neck (P= 0.021) and lumbar spine (P< 0.001), while it is of no statistical significance in total left hip (P= 0.069).

#### 4.4.2. T-Score

Two RCTs [15, 28] reported the changes of T-score. The T-score of Kotlarczyk 2012 [15] was measured by ultrasound, while that of Maria 2017 [28] was gauged by DXA. Due to the substantial heterogeneity (Chi^2^=7.97, Tau^2^=0.02, I^2^=74.9%, and P=0.019), the statistical analysis was abandoned according to the Cochrane Handbook for Systematic Reviews of Interventions [31]. Kotlarczyk 2012 [15] showed that the difference between trial group and placebo group was of no statistical significance in calcaneus (P-value is not available). Maria 2017 [28] also showed that the difference of T-score between trial group and placebo group was statistically significant in the left femoral neck (P< 0.05) and lumbar spine (P< 0.001), while it is of no statistical significance in total left hip (P=0.069).

### 4.5. Secondary Outcomes

**Figure 3:**
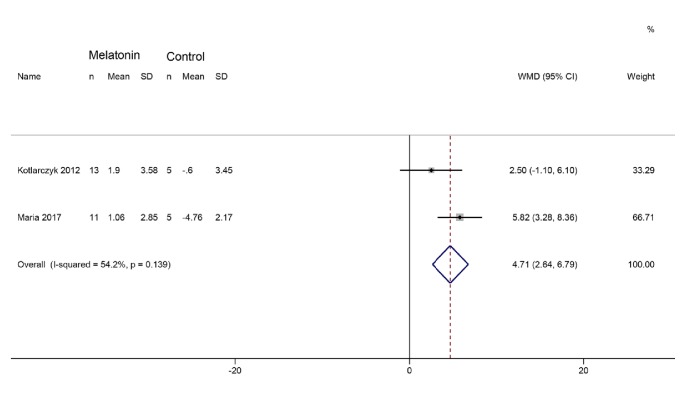
Osteocalcin.

Two RCTs [15, 28] reported osteocalcin. The heterogeneity was medium among RCTs (I^2^= 54.2%, P=0.139); thus the fixed effect model was utilized. The summary result shows that the difference between two groups has statistical significance (WMD 4.97; 95% CI 3.14, 6.79; P < 0.00001) (Figure 3).

### 4.6. Adverse Events

Only two studies [26–28] reported AEs. Maria 2017 [28] showed that no related adverse events occurred in both two groups. Amstrup 2015 [26, 27] reported that the events occurred at equal frequency in both groups.

## 5. Discussions

### 5.1. Main Findings

This research found that melatonin may increase the BMD of the femoral neck and lumbar spine in perimenopausal and postmenopausal women; and combination therapy with melatonin may improve the T-scores of femoral neck and lumbar spine. The meta-analysis also found that melatonin may increase the osteocalcin in perimenopausal and postmenopausal women. Although the difference of the BMD and T-score of total hip was of no statistical significance, Maria 2017 thought that the BMD in total hip of the trial group showed an improvement trend compared with the placebo group [28]. For safety, no obvious adverse events were found in three RCTs.

### 5.2. Overall Completeness and Applicability of Evidences

For primary outcomes, the interventions of RCTs are not the same: the prescription used in Amstrup 2015 [26, 27] is “Melatonin 1 or 3 mg+ Calcium 800 mg+ Vit D3 20 *μ*g,” which is “Melatonin 5 mg+ Strontium (citrate) 450 mg+ Vitamin D3 2000 IU+ Vitamin K2 60*μ*g” in Maria 2017 [28]; in the study of Kotlarczyk 2012 [15], the intervention group was only given melatonin 3 mg oral. The control groups of the three are as follows: “Placebo+ Calcium 800 mg+ Vit D3 20 *μ*g” [26, 27], “Placebo” [28], and “Placebo (Lactose)” [15]. Therefore, Amstrup 2015 [26, 27] and Kotlarczyk 2012 [15] mainly compare the efficacy of melatonin with that of placebo, while Maria 2017 [28] compares the efficacy of melatonin combination preparation with that of placebo. This affects the applicability of the results. Meanwhile, in T-score outcome, two included RCTs utilized different methods for measurement, which also affects the applicability. In addition, the compartment measured when performing the T-score is different, so it cannot accurately reflect the effect of melatonin on the bone of a certain compartment. Last but not least, all RCTs come from Europe and America (Amstrup 2015 [26, 27] comes from Denmark while Kotlarczyk 2012 [15] and Maria 2017 [28] come from America), which may make the results mainly reflect the efficacy of melatonin in Europe and America rather than other regions.

### 5.3. Interpretation of the Outcomes

Melatonin is an endogenous factor secreted by the pineal [14]. The effect of melatonin on osteopenia may be related to its ability to regulate sleep rhythm [33]. Research has shown that the low BMD of postmenopausal women is closely related to poor sleep quality, especially long sleep duration and frequent daytime nap [33]. Melatonin can keep the rhythm of the bone in sync with the light/dark cycle so as to prevent bone loss and osteoporosis [28, 34]. The positive effects of melatonin on mood and sleep may also be achieved by lowering cortisol levels [34]. Melatonin may also affect osteoblastogenesis and osteoclastogenesis through molecules such as MT2 melatonin receptors, MEK1/2, and MEK5, thereby preventing the bone loss [34].

The improvement of bone health by melatonin may also be achieved by stabilizing body weight fluctuations. In the study of Maria 2017 [28], the trial group had a smaller weight difference throughout the year than the placebo group (p = 0.032). New research shows that significant changes in body weight can lead to increased bone turnover and decreased bone mass [35]; mature women with a BMI below 18 kg / m^2^ are estimated to have a higher rate of bone loss than normal women of the same age [36]. A study found that women who took melatonin after menopause have reduced total fat mass and increased lean mass [37]. Another study shows that melatonin inhibits lipogenesis and enhances osteogenesis of human mesenchymal stem cells by inhibiting PPAR*γ* expression and enhancing Runx2 expression [38]. This partly explains the effect of melatonin on bone.

### 5.4. The Strengths of This Review

This is a registered and newest systematic review and meta-analysis that first evaluates the efficacy and safety of melatonin for perimenopausal and postmenopausal women with osteopenia. The included RCTs were carefully assessed for risk of bias and were rated as low bias risk, indicating that the included RCTs are of high quality.

### 5.5. The Limitations of This Review

There are still some problems that affect the applicability of the results (see Section 5.2). Although the quality of RCTs is high, the quantity of RCTs and participants are still not enough (only 3 RCTs and totally 121 patients). Meanwhile, the heterogeneity of BMD and T-scores are too high to undergo meta-analysis. The heterogeneity may come from the potential discrepancies in the pharmacological effects of various melatonin preparations which may result from different standardization of melatonin manufacturing process, dosage, duration of treatment, units of laboratory tests, and races of the selected patients or other places. Another possible source is that the measurement methods and measurement compartments taken by different RCTs are not the same. For example, the T-score of Kotlarczyk 2012 [15] was measured by ultrasound, while that of Maria 2017 [28] was gauged by DXA. The compartment measured by Kotlarczyk 2012 [15] is calcaneus, while that of the other two RCTs is femoral neck, lumbar spine, and so on. Last but not least, the study duration is generally the medium term (6 to 12 months); the long-term efficacy of melatonin is temporarily uncertain.

### 5.6. Implications for Future

The implications for clinical practice are as follows. Melatonin might be used as a safe nutritional supplement to improve bone density in perimenopausal and postmenopausal women based on current evidence, but its efficacy needs to be further affirmed. Melatonin may not only improve bone health but also reduce health care costs of bone disease. In a recent study, the addition of melatonin to osteoporosis treatment prescriptions resulted in significant savings in annual health care costs, reducing the economic burden associated with bone loss therapy [39]. Therefore, more clinical and basic research is needed in the future to clarify whether melatonin can be an early treatment option in the perimenopausal and postmenopausal and age-related bone loss.

The implications for future study are as follows. More RCTs measuring standard compartments (lumbar spine, total hip, and femoral neck) are needed to reduce heterogeneity and get more reliable conclusions. RCTs in the future are also expected to use a standard method (DXA-scans) to measure BMD and T-score, which facilitates statistical analysis in meta-analysis to obtain more reliable results. Meanwhile, future RCTs coming from different countries and regions with more outcomes are expected so as to enlarge the quantity of participants and extend the applicability of the findings. In addition, RCTs with longer follow-up duration are needed to evaluate the long-term efficacy of melatonin. Last but not least, more RCTs observing and reporting adverse events are still in demand so as to thoroughly evaluate the security of melatonin.

## Figures and Tables

**Table 1:** Search strategy for PubMed.

Database	Search strategy
PubMed	Melatonin AND (Perimenopausal Bone Loss OR Bone Loss, Postmenopausal OR Bone Losses, Postmenopausal OR Postmenopausal Bone Losses OR Osteopenia, Postmenopausal OR Osteoporosis, Postmenopausal OR Osteopenia, Postmenopausal OR Postmenopausal Osteoporosis OR Postmenopausal Osteopenia OR Postmenopausal Osteopenia OR Osteoporosis, Postmenopausal OR Postmenopausal Osteoporosis OR Bone Loss, Perimenopausal OR Bone Losses, Perimenopausal OR Perimenopausal Bone Losses OR Postmenopausal Bone Loss) AND (randomized controlled trial [pt] OR controlled clinical trial [pt] OR placebo [tiab] OR drug therapy [sh] OR trial [tiab] OR groups [tiab] OR clinical trials as topic [mesh: noexp] OR Clinical Trial OR random*∗* [tiab] OR random allocation [mh] OR single-blind method [mh] OR double-blind method [mh] OR crossover studies) NOT (animals [mh] NOT humans [mh])

**(a):** 

Study	Journal	Country	Design	Sample size		Mean age (years)		Relevant outcomes
				Trial group	Control group	Trial group	Control group	
Amstrup 2015 [26, 27]	J Pineal Res/ Nutr J	Denmark	RCT, DB	40	41	62.4 ± 3.5	62.9 ± 4.7	BMD, adverse events
Kotlarczyk 2012 [15]	J Pineal Res	USA	RCT, DB	13	5	50.3 ± 3.0	47.5 ± 2.0	T-score, osteocalcin
Maria 2017 [28]	Aging	USA	RCT, DB	11	11	60 ± 1.73	57 ± 1.41	BMD, T-score, osteocalcin, adverse events

RCT: randomized controlled trial; DB: double-blind.

**(b):** 

Study	Inclusion criteria	Exclusion criteria	Identifier	Duration
Amstrup 2015 [26, 27]	(1) Postmenopausal women between 55 and 75 years (2) Osteopenia verified by DXA-scans of total hip or lumbar spine (T-score between -1 and -2.5) (3) Written informed consent after oral and written information	(1) Severely impaired renal function (plasma creatinine >60 eGFR ml/l) (2) Severely impaired hepatic function (plasma alanine aminotransferase (ALAT) and/or alkaline phosphatase more than the doubled compared to upper limit of reference value) (3) Coagulation factors PP <0.6 (4) Hypercalcemia (p-ion calcium > 1.32 nmol/l) (5) Previous or present malignancies (except a treated skin cancer that is not melanoma or treated carcinoma in situ, 2 years since the last therapy) (6) Diseases affecting the calcium homeostasis including untreated thyroid diseases (7) Regular use of medicine affecting the calcium homeostasis, including diuretics, lithium, antiepileptics, and glucocorticoids (8) SSRI-product with fluvoxamine (9) Treatment with carbamazepine (10) Treatment with rifampicin (11) Severe malabsorption syndrome including gastric or intestinal resection (12) Alcohol or drug abuse (13) Smokers (14) Major medical or social problems that will be likely to preclude participation for one year	NCT01690000	12 months
Kotlarczyk 2012 [15]	(1) Women of age 45 or older (2) Experiencing an irregular menstrual cycle (3) Having had at least one menstrual period in the past 6 months	(1) Current use of hormone therapy or birth control (2) Current use of prescription medications for thinning bones, sleep, depression, or regulation of blood pressure (3) Use of medication for thinning bones within the past three months (4) Current use of steroid medications or chronic use in the past 6 months (diagnosis of osteoporosis) (5) Uncontrolled high blood pressure, liver disease, and medical conditions such as hyperparathyroidism or cancer, severe sleep apnea, chronic obstructive pulmonary disease, and severe lactose intolerance (6) Current use of tobacco	NCT01152580	6 months
Maria 2017 [28]	(1) Being postmenopausal with osteopenia (T-score between -1 and -2.5) (2) Willingness to participate in a 12-month study (3) Willingness to take daily therapy right before bed (4) Willingness to undergo testing of bone markers and other biochemical parameters (5) Providing a self-assessment on quality of life throughout the program	(1) Being diagnosed with osteoporosis (T-score less than -2.5) (2) Being osteopenic as a consequence of other medical conditions such as hyperparathyroidism, metastatic bone disease, multiple myeloma, or chronic steroid use (3) Current use of hormone therapy or birth control (4) Current use of prescription medications for osteoporosis, sleep, depression, anxiety, ulcerative colitis, or regulation of blood pressure (5) Current use of steroid medications or chronic use in the past 6 months (6) Other medical conditions such as uncontrolled high blood pressure, liver disease, severe sleep apnea, and chronic obstructive pulmonary disease (7) Current use of tobacco	NCT01870115	12 months

**Table 3:** Oral supplement composition.

Study	Intervention		Medicine preparation
	Trial group	Control group	
Amstrup 2015 [26, 27]	Melatonin 1 or 3 mg+ Calcium 800 mg+ Vit D3 20 *μ*g	Placebo+ Calcium 800 mg+ Vit D3 20 *μ*g	Skanderborg Pharmacy (Denmark)
Kotlarczyk 2012 [15]	Melatonin 3 mg	Placebo (Lactose)	Avalon Pharmacy (Avalon, PA, USA)
Maria 2017 [28]	Melatonin 5 mg+ Strontium (citrate) 450 mg+ Vitamin D3 2000 IU+ Vitamin K2 60*μ*g	Placebo	Pure Encapsulations, Inc. (Sudbury, MA, USA).

## Supplementary Materials

Supplementary 1 “CRD42018086238” is the protocol of this systematic review and meta-analysis.

Supplementary 2 “PRISMA 2009 checklist” is to ensure that this systematic review and meta-analysis follows the PRISMA guidelines.
